# A novel inflammation-based prognostic score for patients with esophageal squamous cell carcinoma: the c-reactive protein/prognostic nutritional index ratio

**DOI:** 10.18632/oncotarget.11389

**Published:** 2016-08-19

**Authors:** Sheng Chen, Xun Yang, Ji-Feng Feng

**Affiliations:** ^1^ Department of Thoracic Surgery, Zhejiang Cancer Hospital, Hangzhou, P.R.China; ^2^ Key Laboratory Diagnosis and Treatment Technology on Thoracic Oncology, Hangzhou, P.R.China

**Keywords:** esophageal squamous cell carcinoma (ESCC), c-reactive protein (CRP), prognostic nutritional index (PNI), cancer-specific survival (CSS)

## Abstract

**Background:**

Inflammation plays a critical role in cancer prognosis. In the current study, we proposed a novel inflammation-based prognostic score, named c-reactive protein/prognostic nutritional index ratio (CRP/PNI ratio), for predicting the prognosis for patients with resectable esophageal squamous cell carcinoma (ESCC).

**Results:**

The optimal cut-off value was 0.10 for CRP/PNI ratio according to the ROC curve. Patients with CRP/PNI ratio ≤0.10 had a significantly better 5-year CSS compared to CRP/PNI ratio >0.10 (44.5% vs. 15.7%, *P*<0.001). On multivariate analyses, we revealed that CRP/PNI ratio was a significant predictive factor of CSS (*P*=0.009). A nomogram could be more accuracy for CSS. The Harrell's c-index for CSS prediction was 0.688.

**Materials and Methods:**

A total of 308 patients with resectable ESCC were enrolled in this retrospective study. The optimal cuf-off value for CRP/PNI ratio was calculated by a receiver operating characteristic (ROC) curve. Kaplan-Meier methods were used to analyse the cancer-specific survival (CSS). Univariate and multivariate analyses were evaluated for CSS. A nomogram was also established to predict the prognosis for CSS.

**Conclusion:**

The CRP/PNI ratio is a novel and useful prognostic score for CSS in patients with resectable ESCC.

## INTRODUCTION

Esophageal cancer (EC) is one of the most common cancers, leading to over 406,800 deaths worldwide and more than 200,000 deaths in China every year [1, 2]. There are two major histological types of EC: squamous cell carcinoma (SCC) and adenocarcinoma (AC) [3]. The predominant pathological type in China is esophageal squamous cell carcinoma (ESCC), which covers more than 90% of all cases [3, 4]. Radical esophagectomy remains the treatment of choice, however, the prognosis is still poor. Therefore, it is important to detect simple and effective biomarkers regarding prognosis for patients with ESCC.

It has increasingly been recognized that inflammation plays a critical role in cancer [5, 6]. Cancer-related inflammation can influence tumor cell migration, invasion and metastasis [6]. Therefore, several inflammation-based hematological biomarkers, such as C-reactive protein (CRP), Glasgow prognostic score (GPS) and prognostic nutritional index (PNI) have been analysed in various cancers [7–11]. However, few studies regarding these inflammation-based biomarkers in patients with EC are available, and the prognostic values of these biomarkers remain uncertain [12–15].

As mentioned above, previous reports have indicated that both CRP and PNI are related to cancer prognosis. However, to our knowledge, no study so far has assessed the clinical significance of the CRP/PNI ratio in other cancers as well as EC. In the current study, therefore, we aimed to evaluate the prognostic role of CRP/PNI ratio for patients with resectable ESCC. In addition, we attempt to establish a predictive nomogram to predict the survival prediction in patients with ESCC.

## RESULTS

**Figure 1:**
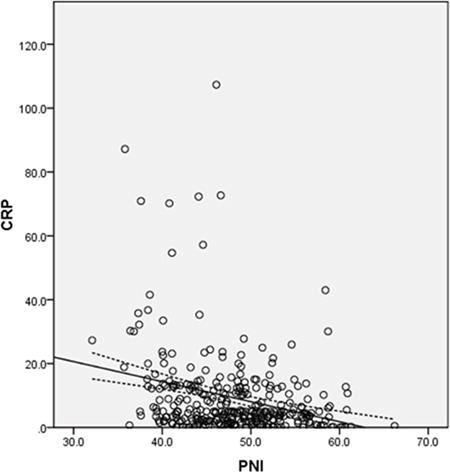
Pearson correlation A significant negative correlation between CRP and PNI (r=−0.279, *P*<0.001).

Among the 308 patients, 40 (13.0%) were women and 268 (87.0%) were men. The mean CRP and PNI were 9.4 ± 13.5 mg/l and 48.0 ± 6.0, respectively. In addition, a significant negative correlation between CRP and PNI was found (r=−0.279, *P*<0.001; Figure 1).

**Figure 2:**
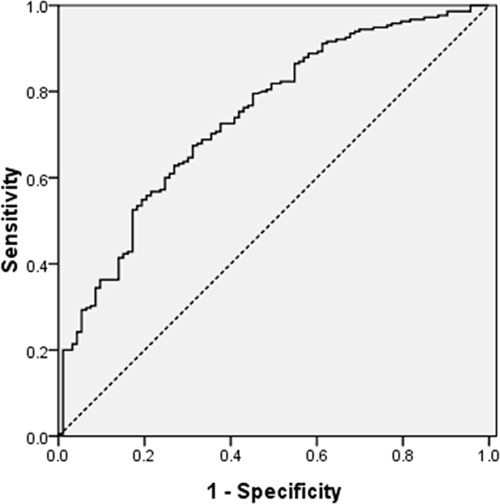
ROC curve for CSS prediction A ROC curve for CSS prediction was plotted to verify the optimal cuf-off value for CRP/PNI ratio, which was 0.10. It demonstrated that CRP/PNI ratio predicts cancer prognosis with a sensitivity of 60.9% and a specificity of 74.2%.

A ROC curve for CSS prediction was plotted to verify the optimal cuf-off value for CRP/PNI ratio, which was 0.10 (Figure 2). It demonstrated that CRP/PNI ratio predicts cancer prognosis with a sensitivity of 60.9% and a specificity of 74.2%. Then, patients were divided into 2 groups: patients with CRP/PNI ratio ≤0.10 and patients with CRP/PNI ratio >0.10. There were 155 (50.3%) patients with CRP/PNI ratio ≤0.10 and 153 (49.7%) patients with CRP/PNI ratio >0.10. The relationships between the CRP/PNI ratio and clinical characteristics were shown in Table 1. Our study revealed that CRP/PNI ratio was associated with tumor length (*P* <0.001), TNM stage (*P* = 0.012), GPS (*P* <0.001), CRP (*P* <0.001), PNI (*P* <0.001), NLR (*P* <0.001) and PLR (*P* <0.001).

**Table 1:** The relationship between CRP/PNI ratio and clinical characteristics

	Cases (n)	CRP/PNI	*P*-value	CRP/PNI		*P*-value
		(mean ± SD)		≤ 0.10 (n)	> 0.10 (n)	
Age (years)			0.399			0.806
≤ 60	175	0.22 ± 0.37		87	88	
> 60	133	0.19 ± 0.25		68	65	
Gender			0.125			0.768
Female	40	0.14 ± 0.15		21	19	
Male	268	0.22 ± 0.34		134	134	
Tumor length (cm)			0.005			<0.001
≤ 3.0	82	0.12 ± 0.28		55	21	
> 3.0	226	0.24 ± 0.33		100	126	
Tumor location						0.348
Upper	17	0.28 ± 0.57	Reference	7	10	
Middle	144	0.22 ± 0.32	0.550	68	76	
Lower	147	0.19 ± 0.29	0.275	80	67	
Vessel invasion			0.709			0.057
Negative	258	0.21 ± 0.33		136	122	
Positive	50	0.23 ± 0.28		19	31	
Differentiation						0.266
Well	44	0.21 ± 0.39	Reference	25	19	
Moderate	204	0.20 ± 0.29	0.757	105	99	
Poor	60	0.25 ± 0.39	0.602	25	35	
TNM stage						0.012
I	73	0.11 ± 0.11	Reference	47	26	
II	104	0.20 ± 0.32	0.005	52	52	
III	131	0.28 ± 0.39	<0.001	56	75	
GPS						<0.001
0	179	0.07 ± 0.07	Reference	134	45	
1	91	0.32 ± 0.36	<0.001	18	73	
2	38	0.52 ± 0.59	<0.001	3	35	
CRP (mg/l)			<0.001			<0.001
≤ 10.0	205	0.07 ± 0.05		153	52	
> 10.0	103	0.49 ± 0.43		2	101	
PNI			<0.001			<0.001
≤ 45	106	0.33 ± 0.43		38	68	
> 45	202	0.15 ± 0.23		117	85	
NLR			<0.001			<0.001
≤ 3.50	203	0.15 ± 0.21		120	83	
> 3.50	105	0.33 ± 0.45		35	70	
PLR			<0.001			<0.001
≤ 150	173	0.14 ± 0.19		103	70	
> 150	135	0.30 ± 0.42		52	83	

**Figure 3:**
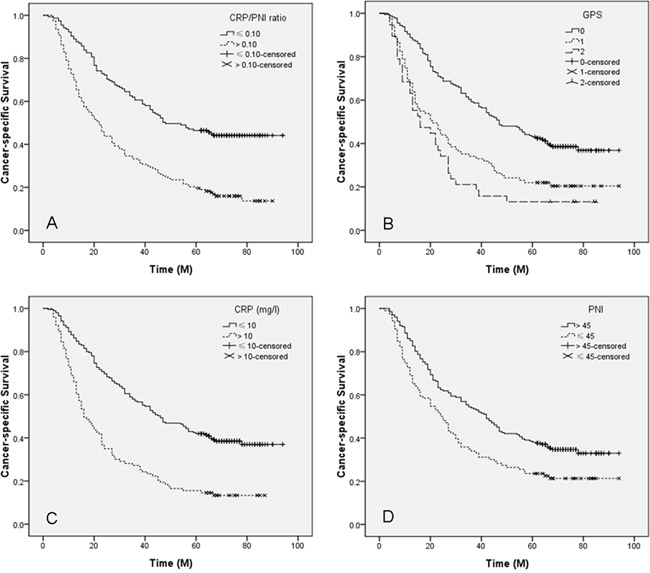
Kaplan-Meier CSS curves stratified by CRP/PNI ratio A., GPS B., CRP C. and PNI D Patients with CRP/PNI ratio ≤0.10 had a significantly better 5-year CSS than patients with CRP/PNI ratio >0.10 (44.5% vs. 15.7%, *P* <0.001). The 5-year CSS for patients with GPS0, 1 and 2 were 38.5%, 20.9% and 13.2%, respectively (*P* <0.001). Patients with elevated CRP (13.6% vs. 38.5%; *P* <0.001) or decreased PNI (21.7% vs. 34.7%; *P* <0.001) were also significantly associated with decreased 5-year CSS, respectively.

**Figure 4:**
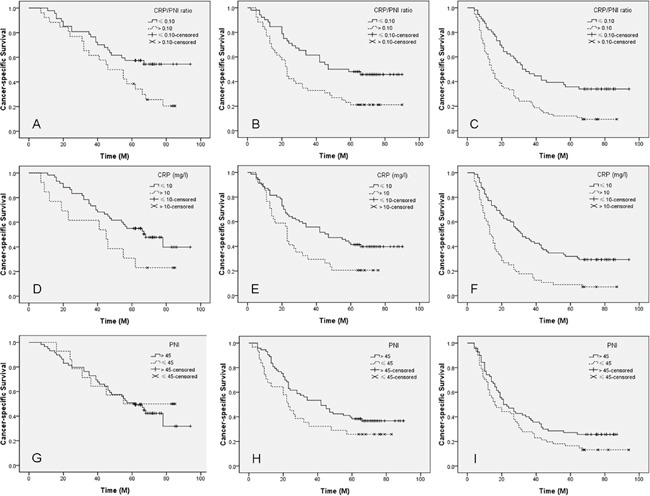
Kaplan-Meier CSS curves stratified by TNM stage CRP/PNI ratio **A-C.** was significantly correlated with CSS based on TNM stage, which was superior to CRP **D-F.** or PNI **G-I.** Patients with CRP/PNI ratio ≤0.10 had a significantly better 5-year CSS than patients with CRP/PNI ratio >0.10 in TNM I (55.3% vs. 23.1%, *P* =0.027; A), TNM II (46.2% vs. 21.2%, *P* =0.003; B) and TNM III (33.9% vs. 9.3%, *P* <0.001; C). CRP was not significantly correlated with CSS in TNM I (48.3% vs. 23.1%, *P* =0.058; D), but significantly correlated with CSS in TNM II (40.0% vs. 20.6%, *P* =0.018; E) and TNM III (29.3% vs. 7.1%, *P* <0.001; F). PNI were not significantly correlated with CSS in TNM I (42.4% vs. 50.0%, *P* =0.686; G), TNM II (37.0% vs. 25.8%, *P* =0.087; H) or TNM III (25.7% vs. 13.1%, *P* =0.076; I).

Patients with CRP/PNI ratio ≤ 0.10 had a significantly better 5-year CSS than patients with CRP/PNI ratio > 0.10 (44.5% vs. 15.7%, *P* <0.001) (Figure 3A). The 5-year CSS for patients with GPS0, 1 and 2 were 38.5%, 20.9% and 13.2%, respectively (*P* <0.001; Figure 3B). In addition, our study revealed that patients with elevated CRP (13.6% vs. 38.5%; *P* <0.001; Figure 3C) or decreased PNI (21.7% vs. 34.7%; *P* <0.001; Figure 3D) were also significantly associated with decreased 5-year CSS, respectively. In subgroup analyses, we demonstrated that CRP/PNI ratio was also significantly correlated with CSS based on TNM stage, which was superior to CRP or PNI (Figure 4).

In univariate analyses, tumor length (*P* =0.029), vessel invasion (*P* =0.011), TNM stage (*P* < 0.001), CRP/PNI ratio (*P* <0.001), GPS (*P* <0.001), CRP (*P* <0.001), PNI (*P* =0.002), NLR (*P* <0.001) and PLR (*P* <0.001) were significant predictors of CSS (Table 2). In multivariate analyses, we demonstrated that CRP/PNI ratio was an independent prognostic factor in patients with resectable ESCC. Patients with CRP/PNI ratio >0.10 had a hazard ratio (HR) of 1.652 [95% confidence interval (CI): 1.131-2.414, *P* =0.009] for CSS. In addition, PLR was also a significant independent predictor of CSS (*P* =0.007). However, the results of our study showed that CRP/PNI ratio was superior to CRP (HR = 1.237, *P* = 0.355) or PNI (HR = 1.055, *P* = 0.761) as a predictive factor in patients with ESCC.

**Table 2:** Univariate and multivariate analyses for patients with ESCC

	CSS	P-value	Univariate	P-value	Multivariate	P-value
			HR (95% CI)		HR (95% CI)	
Age (years)		0.691		0.694		0.700
≤ 60	30.3		Reference		Reference	
> 60	30.1		1.056 (0.806-1.383)		1.056 (0.799-1.396)	
Gender		0.473		0.477		0.887
Female	35.0		Reference		Reference	
Male	29.5		1.160 (0.770-1.748)		0.970 (0.632-1.486)	
Tumor length (cm)		0.027		0.029		0.263
≤ 3.0	36.6		Reference		Reference	
> 3.0	27.9		1.416 (1.036-1.936)		0.814 (0.567-1.168)	
Tumor location		0.644		0.652		0.863
Upper	41.2		Reference		Reference	
Middle	29.2		1.355 (0.708-2.595)	0.359	1.202 (0.616-2.344)	0.590
Lower	29.9		1.341 (0.701-2.568)	0.376	1.193 (0.610-2.335)	0.606
Vessel invasion		0.010		0.011		0.485
Negative	32.6		Reference		Reference	
Positive	18.0		1.556 (1.106-2.188)		1.140 (0.790-1.645)	
Differentiation		0.120		0.128		0.080
Well	40.9		Reference		Reference	
Moderate	28.9		1.290 (0.849-1.958)	0.233	1.271 (0.815-1.981)	0.290
Poor	26.7		1.637 (1.008-2.660)	0.046	1.741 (1.041-2.913)	0.035
TNM stage		<0.001		<0.001		0.001
I	43.8		Reference		Reference	
II	33.7		1.518 (1.031-2.235)	0.034	1.522 (1.006-2.303)	0.047
III	19.8		2.346 (1.632-3.372)	<0.001	2.465 (1.542-3.940)	<0.001
Adjuvant therapy		0.472		0.477		0.110
No	30.2		Reference		Reference	
Yes	30.1		1.112 (0.831-1.487)		0.758 (0.539-1.065)	
CRP/PNI ratio		<0.001		<0.001		0.009
≤ 0.10	44.5		Reference		Reference	
> 0.10	15.7		2.293 (1.742-3.018)		1.652 (1.131-2.414)	
GPS		<0.001		<0.001		0.531
0	38.5		Reference		Reference	
1	20.9		1.910 (1.417-2.574)	<0.001	1.258 (0.839-1.887)	0.266
2	13.2		2.599 (1.755-3.849)	<0.001	1.182 (0.644-2.171)	0.589
CRP (mg/l)		<0.001		<0.001		0.355
≤ 10	38.5		Reference		Reference	
> 10	13.6		2.333 (1.773-3.071)		1.237 (0.788-1.942)	
PNI		0.001		0.002		0.761
> 45	34.7		Reference		Reference	
≤ 45	21.7		1.562 (1.186-2.057)		1.055 (0.747-1.490)	
NLR		<0.001		<0.001		0.949
≤ 3.50	36.5		Reference		Reference	
> 3.50	18.1		1.740 (1.323-2.289)		0.989 (0.713-1.373)	
PLR		<0.001		<0.001		0.007
≤ 150	38.2		Reference		Reference	
> 150	20.0		1.793 (1.371-2.346)		1.505 (1.119-2.025)	

**Figure 5:**
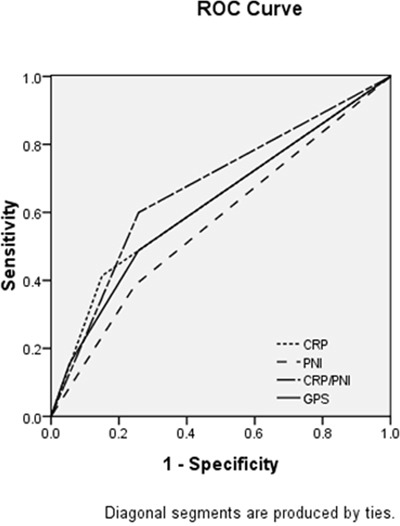
Comparison of the AUC for ROC curves The AUC of the CRP/PNI ratio was higher than other inflammation-based biomarkers, indicating that the CRP/PNI ratio was superior to the GPS, CRP or PNI for prognosis.

The areas under the curve (AUC) was 0.671 (95% CI: 0.606-0.736, *P* <0.001) for CRP/PNI ratio, 0.632 (95% CI: 0.567-0.696, *P* <0.001) for CRP, 0.622 (95% CI: 0.556-0.687, *P* = 0.001) for GPS and 0.569 (95% CI: 0.501-0.638, *P* =0.053) for PNI. The discrimination ability of the CRP/PNI ratio was higher than other inflammation-based biomarkers, indicating that the CRP/PNI ratio was superior to the GPS, CRP or PNI (Figure 5).

**Figure 6:**
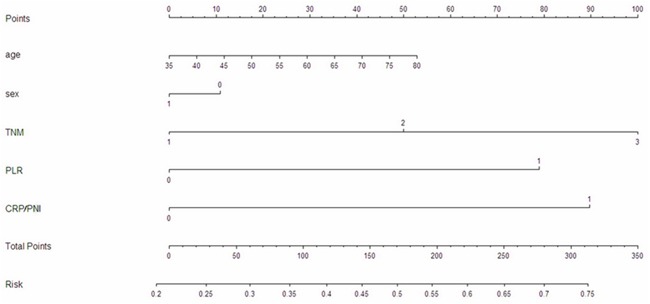
Nomogram model for death risk prediction The nomogram is used by totalling the points identified at the top of the scale for each independent factor. The Harrell's c-index for CSS prediction was 0.688.

To predict the risk for patients with ESCC, a novel nomogram model was established by prognostic factors (TNM stage, PLR and CRP/PNI ratio) combined with age and sex (Figure 6). It can predict the probability of death for patients with ESCC. The Harrell's c-index for CSS prediction was 0.688.

## DISCUSSION

In the present study, a novel inflammation-based prognostic score (CRP/PNI ratio) was conducted based on CRP and PNI and was shown to be an independent predictor for patients with resectable ESCC. To the best of our knowledge, this is the first study to determine the prognostic value of CRP/PNI ratio in predicting prognosis for patients with resectable ESCC.

There is strong linkage between inflammation and cancer. CRP was initially identified as a substance reacting with pneumococcal C-polysaccharide, which appeared in inflammation [16]. Previous published studies have shown that serum CRP is a predictor of survival in several cancers, including EC [7, 8, 12, 13]. A meta-analysis conducted by Huang et al. [17] revealed high levels of CRP were significantly associated with poor survival in patients with EC. In our study, patients with CRP ≤10.0 mg/l had a significantly better 5-year CSS than patients with CRP >10.0 mg/l (38.5% vs. 13.6%, *P*<0.001). However, CRP was not an independent prognostic factor in multivariate analyses (*P*=0.355).

The PNI is calculated based on the serum albumin and lymphocyte count. It was originally proposed to assess the perioperative nutritional conditions for patients with gastrointestinal tumors [10]. Recently, the PNI has been shown to be a prognostic marker for various malignancies [10, 11]. However, few studies regarding PNI in patients with EC are available, and the clinical significance and prognostic value of this marker remain uncertain. Nozoe et al. [15] showed that PNI is associated with tumor progression and survival in patients with EC. However, Sun et al. [18] showed that PNI does not correlate with prognosis in patients with ESCC. In the current study, however, PNI was not an independent prognostic factor (*P*=0.761).

As we know, both CRP and PNI are influenced by various non-cancer-related conditions, and the ratio of CRP and PNI could therefore minimise the potential basis. The prognostic value of the CRP/PNI ratio for ESCC patients would be more reliable than the effect of either CRP or PNI. In the current study, therefore, we firstly investigated the prognostic significance of CRP/PNI in assessing the outcomes in ESCC patients. Patients with CRP/PNI ratio ≤0.10 had a significantly better 5-year CSS compared to CRP/PNI ratio >0.10 (44.5% vs. 15.7%, *P*<0.001). On multivariate analyses, we demonstrated that CRP/PNI ratio was a significant predictive factor of CSS (*P*=0.009). Neither in other cancer nor in ESCC had the significance of CRP/PNI been investigated before. To the best of our knowledge, this is the first time that the CRP/PNI ratio has been found to be a predictor of CSS in patients with ESCC.

In the current study, we used the GPS, a well-known inflammatory parameter, in the Cox regression model, while multivariate analyses showed that the CRP/PNI ratio (*P*=0.009), but not GPS (*P*=0.531), was an independent prognostic factor. From this point of view, the CRP/PNI ratio may have additional prognostic value over the GPS with regard to predicting CSS in ESCC patients. In ROC analyses, our findings revealed that the AUC was higher in CRP/PNI ratio (0.671), than GPS (0.622), CRP (0.632) or PNI (0.569), indicated that the CRP/PNI ratio was superior to other inflammation-based prognostic scores in terms of its prognostic ability in patients with ESCC.

It is well know that nomogram could establish a simple graphic representation of a statistical predictive model [19]. Moreover, several reports revealed that nomogram has been shown to be more accurate than the conventional methods for cancer prognosis [20, 21]. In the current study, therefore, we attempt to establish a predictive nomogram to predict the probability that the death risk for ESCC patients based on TNM stage, CRP/PNI ratio combined with age and sex. The nomogram performed well in predicting CSS by c-index (0.688).

The potential limitations of the present study should be acknowledged. Firstly, our study was a retrospective analyses with a short duration of the mean follow-up. Secondly, we excluded patients who had received neoadjuvant treatment, which may have influenced the result. Thirdly, the difference was large between sex ratio in the current study and esophageal cancer epidemiological data, which may have influenced the result. Finally, we initially used a nomogram to predict prognostic value of CRP/PNI ratio in patients with ESCC, however, it should be better to use external study cohort to validate the nomogram. Therefore, larger prospective studies will need to be performed to confirm these preliminary results.

In summary, there was a significant association between the CRP/PNI ratio and clinical characteristics. Based on the results of the current study, we beleve that CRP/PNI ratio is a novel and useful predictive factor for CSS in patients with resectable ESCC.

## MATERIALS AND METHODS

Between January 2005 and December 2008, a retrospective analysis was conducted for patients with histopathologically confirmed ESCC with no distant metastasis (TNM stage I–III). All patients underwent curative esophagectomy and standard lymphadenectomy. The standard surgical approach consisted of the Ivor Lewis procedure and the McKeown procedure. In our institute, the majority of patients underwent two-field lymphadenectomy. Three-field lymphadenectomy was used only if the cervical lymph nodes were thought to be abnormal upon preoperative evaluation. Patients who had received preoperative therapy were excluded. At last, 308 patients were enrolled in our study. In the current study, a cancer-specific survival (CSS) analysis was ascertained. The last follow-up was 30 June 2013. This study was approved by the Ethical Committees of Zhejiang Cancer Hospital (Hangzhou, China). Patients were staged according to the 7th edition of the American Joint Committee on Cancer (AJCC) Cancer Staging [22].

Routine laboratory results were extracted in a retrospective medical records. The GPS was calculated as follows [9, 14]: patients with elevated CRP (>10 mg/l) and hypoalbuminemia (<35 g/l) were assigned to a score of 2. Patients with one or no abnormal value were assigned to a score of 1 or 0, respectively. The PNI was calculated using following the formula: 10 × serum albumin (g/dl) + 0.005 × total lymphocyte count (per mm^3^) [10, 11, 15]. The cut-off value for CRP and PNI were 10 mg/l and 45 according to the previous studies [7, 8, 10–13].

### Statistical analysis

A receiver operating characteristic (ROC) curve for CSS prediction was plotted to verify the optimal cuf-off value for CRP/PNI ratio. Kaplan-Meier methods were used to analyse CSS. The CSS was defined as the time from the cancer diagnosis until occurrence of cancer-related death or the end of follow up. Univariate and multivariate Cox analyses were performed to analyse the prognostic factors. The areas under the curve (AUC) were calculated and compared using the method reported by DeLong et al. [23]. A nomogram model was established and the predictive accuracy was evaluated by Harrell's concordance index (c-index) [19]. All of the tests were two-sided, and *P* <0.05 was considered to be statistically significant. Statistical analysis was conducted with SPSS 17.0 (SPSS Inc., Chicago, IL, USA) and R 3.2.3 software (Institute for Statistics and Mathematics, Vienna, Austria).
